# Population sensitivity of acute flaccid paralysis and environmental surveillance for serotype 1 poliovirus in Pakistan: an observational study

**DOI:** 10.1186/s12879-018-3070-4

**Published:** 2018-04-13

**Authors:** Kathleen M. O’Reilly, Robert Verity, Elias Durry, Humayun Asghar, Salmaan Sharif, Sohail Z. Zaidi, M. Zubair M. Wadood, Ousmane M. Diop, Hiro Okayasu, Rana M. Safdar, Nicholas C. Grassly

**Affiliations:** 1 Medical Research Council Centre for Outbreak Analysis and Modelling, Department of Infectious Disease Epidemiology, School of Public Health, Imperial College London, London, UK; 2 Faculty of Infectious and Tropical Diseases, Centre for Mathematical Modelling of Infectious Diseases, London School of Hygiene & Tropical Medicine, London, UK; 3 World Health Organization Country Office, Islamabad, Pakistan; 4 World Health Organization Eastern Mediterranean Regional Office, Cairo, Egypt; 5 Department of Virology, National Institute for Health, Chak Shahzad, Islamabad, Pakistan; 6 Polio, Emergencies and Country Collaboration Cluster, World Health Organization, Geneva, Switzerland; 7 National Emergency Operation Centre, Ministry of National Health Services, Regulations & Coordination, Islamabad, Pakistan

**Keywords:** Poliomyelitis, Pakistan, Sewage, Multi-state models, Sensitivity

## Abstract

**Background:**

To support poliomyelitis eradication in Pakistan, environmental surveillance (ES) of wastewater has been expanded alongside surveillance for acute flaccid paralysis (AFP). ES is a relatively new method of surveillance, and the population sensitivity of detecting poliovirus within endemic settings requires estimation.

**Methods:**

Data for wild serotype 1 poliovirus from AFP and ES from January 2011 to September 2015 from 14 districts in Pakistan were analysed using a multi-state model framework. This framework was used to estimate the sensitivity of poliovirus detection from each surveillance source and parameters such as the duration of infection within a community.

**Results:**

The location and timing of poliomyelitis cases showed spatial and temporal variability. The sensitivity of AFP surveillance to detect serotype 1 poliovirus infection in a district and its neighbours per month was on average 30.0% (95% CI 24.8–35.8) and increased with the incidence of poliomyelitis cases. The average population sensitivity of a single environmental sample was 59.4% (95% CI 55.4–63.0), with significant variation in site-specific estimates (median varied from 33.3–79.2%). The combined population sensitivity of environmental and AFP surveillance in a given month was on average 98.1% (95% CI 97.2–98.7), assuming four samples per month for each site.

**Conclusions:**

ES can be a highly sensitive supplement to AFP surveillance in areas with converging sewage systems. As ES for poliovirus is expanded, it will be important to identify factors associated with variation in site sensitivity, leading to improved site selection and surveillance system performance.

**Electronic supplementary material:**

The online version of this article (10.1186/s12879-018-3070-4) contains supplementary material, which is available to authorized users.

## Background

Since the launch of the Global Polio Eradication Initiative (GPEI) in 1988, partners of the GPEI and member states have made great strides towards elimination. By December 2015, circulation of wild type poliovirus (WPV) was limited to Pakistan and Afghanistan, and with the implementation of national emergency action plans, strategies specific to each country are being implemented to immunise all children. In Pakistan, poliovirus transmission has challenged the GPEI partners; transmission is focussed within inaccessible regions of the Federally Administered Tribal Areas, and highly mobile populations that migrate between Khyber Pakhtunkhwa and the city of Karachi. In 2014 cases from Pakistan accounted for 85% of the global case count [1].

Surveillance for poliomyelitis relies on investigation and reporting of children who develop acute flaccid paralysis (AFP). As the final stages of poliomyelitis eradication approaches, detection of poliovirus is critical and additional surveillance activities are important. Transmission of poliovirus mostly results in asymptomatic infection, such that approximately 100 to 1000 infections occur for each case [2]. This makes it possible for circulation of poliovirus to occur for a substantial period of time before poliomyelitis cases appear. Indeed, poliomyelitis has been detected in Nigeria after several years of silent transmission that most likely reflects suboptimal surveillance in a politically challenging setting [3]. Additional surveillance activities for poliovirus such as sampling of sewage wastewater, known as environmental surveillance (ES) are helpful to support eradication [4–7]. Poliovirus has been successfully isolated from samples of wastewater from a converging network within several settings, and sometimes in the absence of poliomyelitis cases [6], suggesting that ES is a highly sensitive method of detection. ES in Pakistan and India have identified genetic lineages that are absent from poliomyelitis cases, further supporting the higher sensitivity of detection from sampling wastewater [4, 8].

A theoretical framework for evaluating the sensitivity of surveillance for poliovirus was outlined by Gary et al. [9]. The population sensitivity of a surveillance system was defined as the probability that a person with poliomyelitis will be identified by the system. In 1997 when the original paper was written few countries used environmental surveillance for poliovirus and so the focus was on surveillance for cases of AFP. Now detection of poliovirus circulation (rather than only poliomyelitis cases) is a priority for surveillance. We use the definition of population sensitivity as the probability that a surveillance system (consisting of data, possibly from multiple sources) detects poliovirus within a population that contains at least one infected person. The sensitivity of a surveillance system is thought to depend on several factors that can be grouped into laboratory sensitivity and collection efficiency. Laboratory sensitivity consists of the procedures used to concentrate samples and determine the presence of poliovirus. The standard protocol for ES concentration and testing in Global Polio Laboratory Network laboratories consists of the “2-phase separation method” followed by isolation of poliovirus via inoculation in specific cell lines, and this technique has been shown to be highly sensitive to detection of poliovirus within a sample [5]. Collection efficiency for AFP surveillance is determined by the number and risk profile of AFP cases identified [10]. For ES surveillance collection efficiency will depend on the frequency of collection and the sensitivity of samples from specific sites. A theoretical framework for sewage water surveillance was developed by Ranta et al. [11] and identified that sampling frequency would increase surveillance sensitivity, but quantitative comparisons of surveillance sensitivity has yet to be estimated directly from affected populations. Focussing on site selection, selected sites target areas with high population density and under-vaccinated or high-risk populations. Although poliovirus has been isolated from flushing experiments within systems that may drain sewage a large number of individuals [6, 12], it is unknown how the site sensitivity of ES scales with the prevalence of infection within a catchment population. Sites for sampling are also selected based on the local topography, drainage information, and accessibility of flowing wastewater. Considering the many factors that can influence site sensitivity, it is likely that there is considerable variation in sensitivity and methods are required to identify which factors play a role.

The aims of this analysis are to determine the sensitivity of ES in Pakistan and investigate the extent of variation in sensitivity, through the statistical analysis of data from environmental and AFP surveillance during 2011–2015. We use a simple multi-state model to estimate the changing infection status of districts in time, and from this estimate the population sensitivity of both surveillance systems. Variation in the sensitivity of surveillance was explored by comparing different model structures to the data and examining the association of estimates to available covariates.

## Methods

### Surveillance for poliovirus in sewage and wastewater

**Fig. 1:**
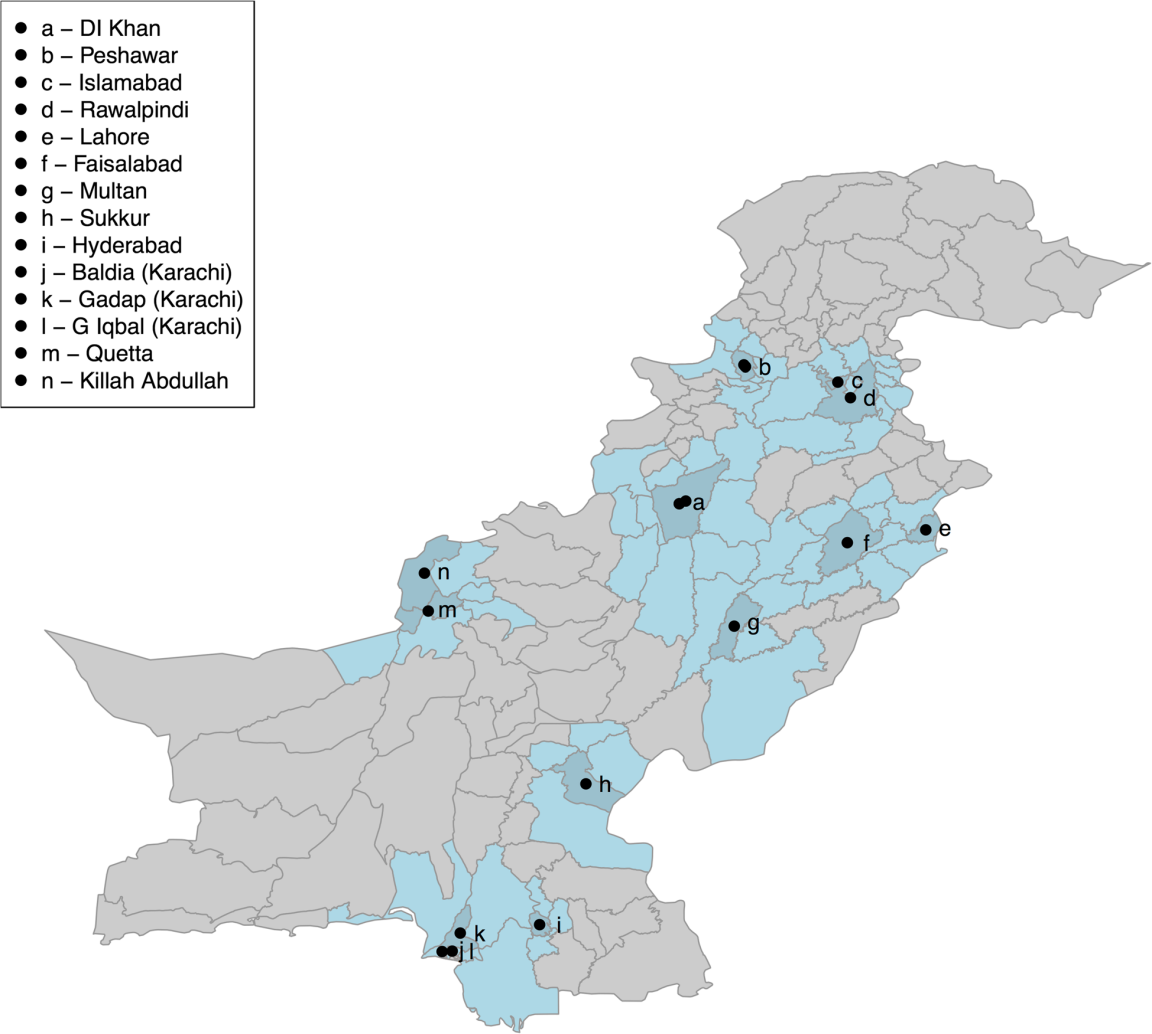
Location of environmental sampling sites in Pakistan. Districts coloured in dark blue the district that sites are located in and the light blue districts are neighbouring districts. The dots are placed in the centre of the district where sampling is carried out; multiple sites are present within some districts

ES was initiated in Pakistan in 2009 at sites within Karachi and Lahore [4]. Sampling has since been expanded to many regions within Pakistan (Fig. 1), focussing on major transportation hubs, and areas with either highly mobile populations or areas associated with suboptimal AFP surveillance or vaccination coverage. The number of sites per district in Pakistan varies from one to three (Table 1), and the rationale behind their selection has been described elsewhere [5]. A majority of the sampling locations are large trenches that contain outlets for sewage and wastewater from communities. Sites in Lahore, Faisalabad and Hyderabad were collected from the inlets of pumping stations.

**Table 1:** Districts within Pakistan where environmental sampling had been initiated between January 2011 – August 2015 and associated information on: neighbouring districts, population size, number of sampling sites, number of samples containing WPV, and cases of poliomyelitis

District	Neighbours	Total population size (‘000)		Environmental sampling				AFP surveillance and poliomyelitis cases			
				Sites	First sampled	Total	WPV^a^ (%)	non-polio AFP rate^b^		Poliomyelitis cases	
		District	Neighbours					District	Neighbours	District	Neighbours
DIKhan	Bhakkar, Dera Ghazi Khan, Lakki Marwat, Layyah, Mianwali, Musakhel, Sharani, Tank, South Waziristan	1233	10,170	3	Oct-14	28	4 (14.3)	11.5	17.4	0	43
Peshawar	Charsada, Khyber, Kohat, Mohmand, Nowshera, Charsada, Khyber, Kohat, Mohmand, Nowshera, Peshawar, Kohat, FR Kohat, FR Peshawar	2556	7097	2	Jan-11	117	85 (72.6)	17.7	24.9	55	593
Islamabad	Haripur, Rawalpindi	1303	6379	1	Mar-14	18	2 (11.1)	2.6	5.6	0	0
Rawalpindi	Abotabad, Attock, Bagh, Chakwal, Haripur, Islamabad, Jhelum, Kotli, Mirpur, Poonch, Sudnuti	3779	17,747	2	Jan-11	76	34 (44.7)	6	7.3	0	4
Lahore	Kasur, Nankana Sahib, Sheikupura	5946	14,529	3	Jan-11	216	67 (31)	6	6.4	1	2
Faisalabad	Hafizabad, Nankanasahib, Okara, Sahiwal, Toba Tek Singh, Jhang, Chiniot	6597	20,274	3	Sep-12	96	2 (2.1)	6.6	8.3	0	4
Multan	Bahawalpur, Khanewal, Lodhran, Muzfargarh	4033	15,157	3	Jan-11	168	33 (19.6)	10	11	0	3
Sukkur	Ghotki, Kashmore, Khairpur, Shikarpur	1113	6302	2	Apr-12	81	17 (21)	10.1	14.5	1	2
Hyderabad	Jamshoro, Matiari, T Allahyar, Thatta, Tando Muhammad Khan	1846	5628	1	Jul-12	38	21 (55.3)	4.6	9.7	0	0
Baldia (Karachi)	Kamari, Orangi, Site	462	2457	2	Jan-11	113	29 (25.7)	9.3	5.6	5	7
Gadap (Karachi)	Jamshoro, Binqasim, Gulshan Iqbal, Gulberg, Kamari, Malir, North nazim, North Karachi, Orangi, Site, Lasbela	429	7633	3	Jan-11	141	75 (53.2)	25	8.1	14	15
Gulshan Iqbal (Karachi)	Gadap, Gulberg, Jamsheed, Lliaqat, Malir, Shahfaisal	1094	5057	2	Jan-11	112	40 (35.7)	7.6	43.2	2	15
Quetta	Killah Abdulah, Mastung, Noshki, Pishin, Ziarat, Harnai	1724	2916	3	Jan-11	147	51 (34.7)	11.1	12.4	27	59
Killah Abdullah	Pishin, Quetta	389	2504	2	Oct-14	22	8 (36.4)	14.6	12.5	11	7

^a^Wild poliovirus isolation (in environmental samples) containing serotype 1 - WPV

^b^per 100,000 children under 15 years old

Samples are collected from each site on a monthly basis. Approximately one litre of sewage is collected using the “grab sample” technique, and in the lab a 500 ml sample is concentrated using a 2-phase separation method and the samples are examined for the presence of poliovirus using a standardised protocol [14, 15]. Positive isolates are further investigated using real-time reverse transcriptase polymerase chain reaction and intratypic differentiation to distinguish between WPV, vaccine-derived poliovirus (VDPV) and Sabin of each serotype and non-polio enteroviruses. We collated and analysed data on serotype-1 WPV between 1st January 2011 to 31st September 2015.

### Surveillance for acute flaccid paralysis

Cases of AFP in all children aged < 15 years and suspected cases of poliomyelitis in persons of any age are reported and investigated as possible cases of poliomyelitis. Faecal samples are collected from affected patients and/or healthy contacts, and if poliovirus is isolated from stool the case is confirmed as poliomyelitis [16]. The majority of AFP cases are not caused by poliovirus [17]. Active AFP surveillance rapidly increased in Pakistan from 2005 to 2010, resulting in an AFP rate of ~ 8 cases per year per 100,000 children under 15 years of age since 2010, which is substantially above the international target of 2 per 100,000 child years. The AFP rate does not directly translate into a quantitative measure of surveillance quality as the true non-polio AFP rate experienced within a population is unknown and depends on the local disease profile and other unknown effects. AFP surveillance is also measured against the percentage of cases that are classified as “adequate” (where at least 2 stool samples per patient are analysed that have been collected at least 24 h apart and within 14 days of onset of paralysis). Summary statistics of the non-polio AFP rate are provided in Table 1 to illustrate that AFP surveillance has been fully functioning within Pakistan during the period of study, and province-level reports of the percentage of adequate stools from AFP cases consistently above 80% [18].

Cases of poliomyelitis caused by serotype-1 WPV with onset of paralysis from 1st January 2011 to 31st September 2015 were used in the analysis. These data were restricted to poliomyelitis cases within districts where environmental sampling was carried out and their immediate neighbours (Table 1).

### Statistical analysis

Both the environmental and AFP surveillance data were aggregated by month, where environmental samples consisted of the number of samples positive for serotype-1 WPV out of the number sampled and AFP data consisted of the number of poliomyelitis cases caused serotype-1 WPV. To enable a fair comparison between surveillance sources, we only analyse data from the start of implementation of ES in each district, where 6 of the 14 districts introduce ES after January 2011. The analysis was carried out considering a) only AFP cases within each district where environmental sampling was carried out, and b) AFP cases within each district and their neighbours. The median duration of consecutive months for positive environmental samples or poliomyelitis cases was estimated with 95% confidence intervals by bootstrap sampling.

**Fig. 2:**
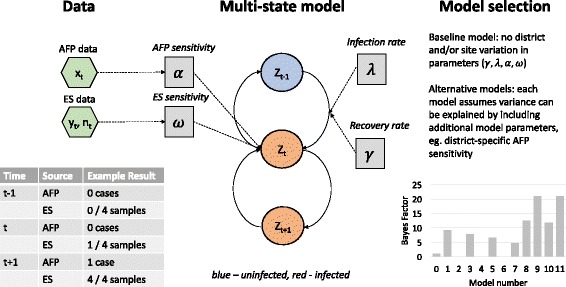
Schematic of the model framework. Inputs into the model (green hexagons) are AFP and ES data from each district each month. The model assumes that a district is either infected or uninfected at each time-point (states are indicated by circles), and transitions (solid arrows) between states are determined by the data and model parameters (grey boxes). Candidate models are compared to the baseline model by estimating the Bayes Factor of each

A multi-state model was used to capture the dynamics of WPV in Pakistan. Multi-state models have been used extensively in the medical literature when time-series data are available and rates of progression through various stages require estimation even if they are not directly observed [19, 20]. Within this framework a district is assumed to be either uninfected or infected and transition between states is controlled by the infection rate (*λ*) and the recovery rate (*γ*). Layered on top of this are the observations, ie. the surveillance data (Fig. 2). For each month if either AFP or ES identifies poliovirus that district is assumed to be infected, corresponding to 100% specificity. If both surveillance sources do not identify poliovirus the district could be either uninfected (ie. a true negative) or infected (ie. a false negative), which will be a function of the probability of being infected and the sensitivity of each surveillance source. We use the definition of population sensitivity (as described in the Introduction) as the probability that a surveillance system detects poliovirus within a population that contains at least one infected person. The parameters of the model, including augmented data of the probability that each time-district observation is positive, are estimated within a Bayesian framework. Full details of the model, including equations, are given in the Additional file 1: Information S1. The sensitivity of each surveillance source is allowed to vary from 0 to 100%, with beta distributed priors (where the mean value was 0.5 and 95% credible intervals 0.1–0.90).

Potential covariates with AFP and ES sensitivity, such as catchment size [21] and incidence of poliomyelitis, were explored. Catchment area estimates are at least 10-fold smaller than population estimates within each district (Additional file 1: Information S2). A log-linear relationship between incidence and surveillance sensitivity was tested and a mixed-effects model with district and site random effects was also specified. Population immunity estimated from non-polio AFP data was not included as an explanatory variable as there was little temporal change in population immunity during the time-period of the analysis. Bayes factors (BF) were used to assess the evidence in favour of each model when compared to the simple model, and may be interpreted in a similar manner to a likelihood ratio, where a BF above 1.00 indicates evidence in favour of the extended model, and larger values indicate stronger evidence. Model parameters were estimated using Markov chain Monte Carlo methods and BFs were calculated by estimating the marginal likelihood [22]. Owing to the complexity of the models (such as the inclusion of augmented data) the marginal likelihood was estimated using thermodynamic integration methods [23] (Additional file 1: Information S3).

Environmental and AFP surveillance sensitivity estimates (denoted by *ω* and *α*, respectively) were combined to estimate the total sensitivity of surveillance within a district using.

$$ \rho =1+\left(\alpha -1\right){\left(1-\omega \right)}^4 $$

assuming four environmental samples per month. The surveillance sensitivity was combined with estimates of the probability of infection in each district (which was estimated in the model) to determine the false omission rate (FOR);

$$ FOR=\frac{\left(1-\rho \right)\Pr (I)}{1-\rho \Pr (I)} $$

where Pr(*I*) is the probability of being infected. The probability of being infected was estimated as part of the parameter estimation.

To establish whether parameter estimates from the multistate model were accurate and precise and to determine whether model selection was prone to type I and type II errors, simulation was used (Additional file 1: Information S4 and Figure S1). All the parameter estimation and model comparisons were carried out using the statistical software R (version 3.2.5.).

## Results

**Fig. 3:**
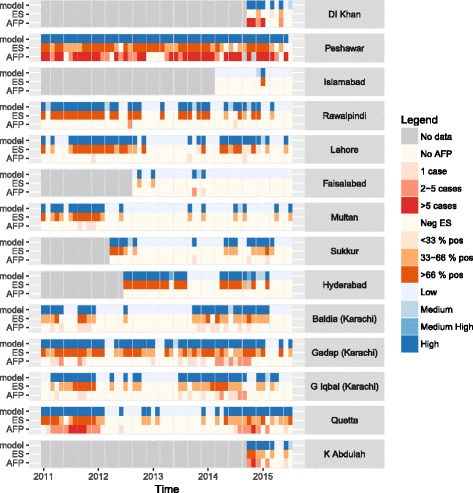
Time-series data and model output for each district included in the analysis, January 2011 to August 2015. AFP cases (red boxes, lower rows) and ES (orange boxes, middle rows) vary in time, and these data can be compared to estimates of the probability that a district is infected (blue boxes, upper rows). Grey areas indicate that environmental sampling had not been initiated within the district

Between 1 January 2011 and 31 August 2015, there were 870 cases of serotype 1 poliomyelitis within the 14 districts and their neighbours where environmental sampling was implemented in Pakistan, and these data are used in the rest of the analysis (Table 1). During this period of time in Pakistan there were large changes in incidence across the country; there were only 58 cases in 2012, and following this an increase was observed in 2014 resulting in 306 cases of wild poliomyelitis. Incidence was also seasonal across the country, peaking in September to October (Fig. 3). A total of 1373 environmental samples were collected from 32 sites within 14 districts in Pakistan, and 468 (34.1%) of samples were positive for serotype 1 WPV (Table 1). All districts reported at least one positive environmental sample, varying in prevalence from 2.2% (Faisalabad) to 72.6% (Peshawar), whereas poliomyelitis cases were only reported from 12 of the 14 districts and their neighbours (four consisted of cases only in the neighbouring districts). The median duration of consecutive months that a district had either positive environmental samples or poliomyelitis cases was 2 months (interquartile range 1–4 months). There was broad agreement in the time-series between surveillance sources (Fig. 3), as indicated by coincident positive months, although there were often positive months of environmental samples that did not correspond with poliomyelitis cases (for example Rawalpindi in 2011 and Hyderabad in 2012–2013).

**Fig. 4:**
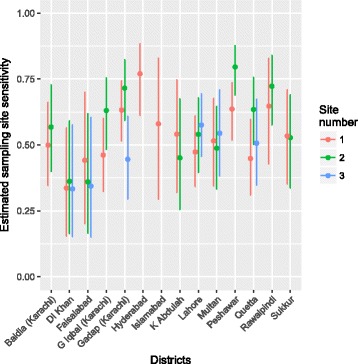
Estimates of environmental site sensitivity for detection of serotype 1 WPV for each district included in the analysis. The number of sites per district varies from 1 to 3. 95% CI are indicated by the vertical lines

**Fig. 5:**
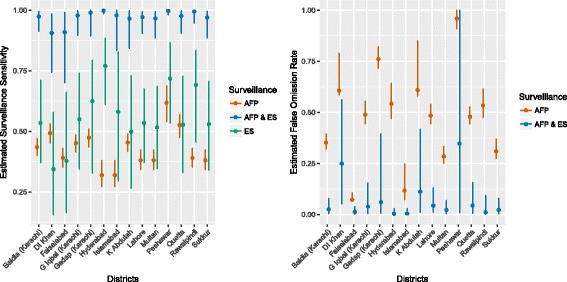
Estimates of the sensitivity of surveillance by each surveillance source (**a**), and the false omission rate (**b**) estimated from the best-fitting multistate model. Vertical lines indicate the 95% credible intervals of the estimate

A model with both variation in ES and AFP surveillance sensitivity had a substantial improvement in model fit when different candidate models were compared to the simplest model with 4 parameters (BF = 21.6 when compared to the simplest model, Table 2). The ES section of the model assumed a mixed-effects structure and the AFP surveillance section of the model assumed a linear increase in sensitivity with poliomyelitis incidence. The site estimates for ES sensitivity are illustrated in Fig 4; the site variance (0.249 (95% CI 0.170–0.385)) was moderately larger in value than district variance (0.177 (95% CI 0.124–0.259)) and the average estimate of sensitivity per site was 53.7% (95% CI 36.1–70.4) (Plots of the prior and posterior distribution are given in Additional file 1: Figure S2). There was insufficient evidence to link estimates of site-specific ES sensitivity to the estimated catchment size of the location of each sampling site. The estimated average duration of poliovirus infection within a district was 3.4 months (95% credible intervals (CI) 2.6–4.3 months) and the rate of infection was 0.42 per month (95% CI 0.32–0.55). The average sensitivity of AFP surveillance was 30.0% (95% CI 24.8–35.8), with considerable variation in sensitivity between districts (Fig 5a). A single environmental sample was estimated to have an average sensitivity of 59.4% (95% CI 55.4–63.0), where 4 samples were estimated to have an average sensitivity of 97.3% (95% CI 96.1–98.1). When four environmental samples (equivalent to a month of environmental sampling per district) and AFP data were combined, the sensitivity of surveillance was 98.1% (95% CI 97.2–98.7) for a district, but variation between districts was apparent (Fig. 5a). The probability that a district was infected varied monthly (Fig. 3); districts such as Peshawar and Quetta were more likely to be infected than not and months with no positive environmental samples or cases have a higher probability of being infected than for other districts. The false omission rate (FOR) varied substantially by district when considering only AFP surveillance, and was high in value when districts were frequently infected (Peshawar and districts within Karachi, Fig 5b). Inclusion of ES increased the sensitivity of detecting poliovirus and consequently reduced the FOR to an average of 3.6%. In the districts of Karachi inclusion of ES reduced the FOR from 40%, 76% and 52% to 2%, 8% and 3% for Baldia, Gadap and Gulshan Iqbal, respectively. For Peshawar the 95% CIs were wide because there were few instances where the district had only negative samples, suggesting a high probability of being infected. When using AFP data from only the district similar model results were obtained but estimates of AFP sensitivity were moderately lower in value. Similar parameter estimates were obtained when the ES sensitivity was considered only at a district level.

**Table 2:** Bayes factors for each model applied to AFP and environmental surveillance data of serotype 1 WPV in Pakistan, January 2010 – August 2015 . A Bayes factor greater than 1.00 indicates an improved model fit when compared to the baseline model

Assumption for AFP surveillance	Assumption for environmental surveillance	Number of parameters	Model evidence	Bayes Factor
One value	One value	4	− 502.9	NA
Linear increase with log_10_(incidence)	One value	5	− 493.9	9*
One value	Linear increase with catchment size	5	−504.5	−1.6
Linear increase with log_10_(incidence)	Linear increase with catchment size	6	− 495.3	7.6*
One value	Quadratic relationship with catchment size	6	− 505.5	−2.6
Linear increase with log_10_(incidence)	Quadratic relationship with catchment size	7	− 496.3	6.6*
One value	Mixed effects structure (no association with catchment size)	6	− 491.4	11.5*
Linear increase with log_10_(incidence)	**Mixed effects structure (no association with catchment size)**	**7**	**− 481.3**	21.6*

The starred models have an improved fit to the data in comparison to the simplest model and the best-fitting model is highlighted in bold

## Discussion

ES for polioviruses will play a crucial role in the eradication of poliomyelitis. A primary aim of ES is the strengthening of surveillance activities by increasing detection of WPV. This study has illustrated in an endemic setting the improved sensitivity when utilising both environmental and AFP surveillance, resulting in a sensitivity of detection of WPV above 90%. The multistate modelling approach indicates some variation in ES sensitivity between sites, but the scale of variation is small.

Several studies have previously shown that wastewater sampling is a highly sensitive method to detect poliovirus within a population shedding poliovirus in stool [7, 13]. These studies were controlled experiments where patients were orally vaccinated with an attenuated monovalent oral polio vaccine and wastewater was collected from a site downstream from the study location. In this analysis we use surveillance data from an endemic setting where the sampling of sites is based on local assessment of the epidemiology, and estimate the sensitivity of surveillance when the infection status of the population is unknown but estimated using a statistical model. The use of “grab samples” are the only feasible method to obtain wastewater samples in a setting such as Pakistan, but are likely to have a reduced sensitivity when compared to 24 h composite samples used in high-income settings where environmental sampling was initially trialled. It is therefore promising that one wastewater sample has in most circumstances a greater ability to detect poliovirus than AFP alone, and in combination with AFP has a substantially improved ability to detect poliovirus. These findings have been corroborated by a combined epidemiologic and genetic analysis of surveillance data within Pakistan [24], illustrating that fewer “orphan viruses” (virus lineages with large gaps in observed sequence mutations) are detected when ES is used in surveillance and that distinct viral lineages are detected more rapidly. The sensitivity of grab samples may be further improved by increasing the volume of water assayed [25], and a field study is on-going to assess the feasibility of a bag-mediated filtration system to collect larger water volumes.

We identified that sensitivity of ES varied, but the reasons for this are unclear. The catchment population size of the sampling sites were estimated using a novel method [21], but do not account for sewage drainage or population movement of residents both inside and outside of the catchment areas. Substantial efforts were made to identify suitable sites for environmental sampling in Pakistan [4], and in most districts multiple sites are used in an attempt to increase the catchment area and target high-risk populations. It may be that the catchment size estimates are not accurate enough to illustrate a relationship, or that other factors (such as the presence of pollutants, variation in wastewater disposal or other unknown factors) play a larger role in determining sensitivity. Consequently, these findings remain only indicative and further study is required. The sensitivity of ES from some districts were comparatively low; within DI Khan and Faisalabad AFP and ES observations were discordant and for Faisalabad genetic sequencing suggested 2 distinct virus lineages detected in AFP and ES. Additionally, environmental sampling had been initiated relatively late in DI Khan and this resulted in wider confidence intervals of the ES sensitivity estimates. A limitation of our approach is that we restrict the analysis to districts located near to environmental sampling sites, rather than apply the analysis to the entire country. In the analysis ES out-performs AFP surveillance but if the findings were extrapolated to the entire country the limited scope of ES is also likely to limit the sensitivity of ES at a national scale. The processing of environmental samples is considerably more costly (in time and use of facilities) than AFP samples and therefore a balance needs to be sought between the use of both surveillance sources [6]. The GPLN recommends 2-phase separation for virus concentration, the use of five flasks in the virus culture step, and separate lab facilities for processing environmental samples [14]. Consequently, even though the ENV samples are much smaller in number than AFP samples the resources required remain substantial [26].

The continued identification of poliomyelitis cases and positive environmental samples from Peshawar indicate the sustained transmission of poliovirus within this region. The frequent population movement between Peshawar and populous areas such as Karachi has enabled reseeding of infection into this and other areas. Vaccination strategies are being implemented to maximise population immunity despite accessibility issues, using house-to-house immunisation activities and more tailored activities such as a short-interval additional dose strategy and community supported vaccination drives [18, 27]. In other areas of Pakistan, ES has shown the presence of poliovirus in the absence of cases, supporting the need to maintain high population immunity through vaccination.

The multistate framework is an ideal method to analyse infectious disease data where multiple data sources are available, as it enables estimation of the combined sensitivity and provides insight on the degree of infection by implicitly estimating the state of the system in time. The framework is very flexible, as transition rates from almost any configuration of states can be calculated, and the framework can be extended to include more complicated models. The model described in this paper is relatively simple, in that it only classifies districts as infected or not, whereas in reality it is likely that districts with low AFP sensitivity are a result of infection only being transient, as opposed to continued transmission observed in other districts (eg. Peshawar). The simplicity of the model ensures that the framework can be applied to other aspects of poliovirus eradication, such as the detection of vaccine-derived polioviruses, and other disease systems where asymptomatic infection and sub-optimal surveillance tools are a concern. We assumed a constant infection rate, which may not be realistic within Pakistan as poliovirus transmission is typically seasonal and variation in the proportion susceptible (due to vaccination) may have influenced the infection rate. Specifying a more complex model (such as varying the infection rate in space and time) may enable more accurate estimates of surveillance sensitivity but comes at a cost of increasing the number of parameters for which the current data may have insufficient power. This aspect of the analysis will be revisited.

## Conclusions

The higher sensitivity of ES when compared to AFP surveillance illustrates that use of ES will improve detection of polioviruses within affected populations. As we approach the final stages of polio eradication, detection of all polioviruses becomes increasingly important, and ES will play an essential role. However, this study highlights several areas of refinement that are required. It is not possible to implement ES in all districts, consequently detection of poliovirus will always require AFP surveillance and estimation of the sensitivity of the total surveillance system within a country is required. This research has also identified considerable variation in the sensitivity of specific sites; further research is required to identify if this variation can be explained. Looking forward, ES will also assist with the phased removal of the different serotypes of the oral poliovirus vaccine, as the high sensitivity of the system will assist in detection of circulating vaccine-derived polioviruses.

## Additional file

Additional file 1: This file contains specifics of the model structure, parameter estimation, model comparison and further details of the data [28–31]. (PDF 2801 kb)

## Abbreviations

AFP: Acute flaccid paralysis
BF: Bayes factors
ES: Environmental surveillance
FOR: False omission rate
GPEI: Global Polio Eradication Initiative
VDPV: Vaccine-derived poliovirus
WPV: Wild type poliovirus
