# Supplementary material for: Population sensitivity of acute flaccid paralysis and environmental surveillance for serotype 1 poliovirus in Pakistan: an observational study
Source: BMC Infect Dis. 2018 Apr 13;18:176. doi: 10.1186/s12879-018-3070-4 (PMC5899327; doi:10.1186/s12879-018-3070-4)
Supplement: Supplementary file 1 — This file contains specifics of the model structure, parameter estimation, model comparison and further details of the data [28–31]. (PDF 2801 kb) [file 12879_2018_3070_MOESM1_ESM.pdf]

Supporting Information

Population Sensitivity of Acute Flaccid Paralysis and Environmental Surveillance for Serotype-1 Poliovirus in Pakistan: an Observational Study

Kathleen M O'Reilly, Robert Verity, Elias Durry, Humayun Asghar, Salmaan Sharif, Sohail Z Zaidi, M Zubair M Wadood, Ousmane M Diop, Hiro Okayasu, Rana M Safdar, Nicholas C Grassly

S1. Description of the multistate modelling framework and parameter estimation

Within each district we assumed the population was either free from poliovirus circulation (uninfected) or infected with poliovirus. These assumptions correspond to a two-state Markov process with constant hazard rates λ and γ of infection and recovery, respectively. A Markov process is one in which the future state of the system depends only on the present state of the system and not on the total history. Using the steps outlined in Gentleman *et al.* [1], the transition probability matrix can be derived for this and more complex systems, and is essentially a function of the hazards λ and γ. A transition probability matrix $P(t)$ is used to represent the probability of moving from one state to another, where p denotes the transition intensity of moving from uninfected to infected and $1-q$ refers to moving from infected to uninfected. For a two-stage Markov model the transition intensities can be easily calculated by solving the differential equations that describe the system;

$$P(t) = \begin{bmatrix} 1-p & p \\ 1-q & q \end{bmatrix} = \begin{bmatrix} \frac{1}{\lambda+\gamma}(\lambda + e^{-(\lambda+\gamma)t}) & \frac{\lambda}{\lambda+\gamma}(1-e^{-(\lambda+\gamma)t}) \\ \frac{\gamma}{\lambda+\gamma}(1-e^{-(\lambda+\gamma)t}) & \frac{1}{\lambda+\gamma}(\lambda+\gamma e^{-(\lambda+\gamma)t}) \end{bmatrix}$$

The state of the system within district $i(i = 1, ..., M)$ at time $t(t = 1, ..., T)$ is denoted by z_{it}, where a value $z_{it} = 0$ denotes an uninfected district and a value $z_{it} = 1$ denotes an infected district. The initial conditions for z (ie. $z_{it=0}$) are estimated. It is important to note that z_{it} is an augmented (latent) parameter, as the true infection status is not directly observed but only estimated through the surveillance data. The AFP data are denoted by x_{it} where if $x_{it} = 0$ then no poliomyelitis cases were reported in the district and if $x_{it} = 1$ then at least 1 poliomyelitis case was reported within the time period and the sensitivity of one AFP month is denoted by α, in other words the probability of a positive result in an infected district, $p(x_{it} = 1|z_{it} = 1)$, is equal to α. The environmental data consist of y_{sit} positive samples from s sites within each district, from a total of n_{sit} samples tested that month, with sensitivity ω per sample. In the first instance we assume that ω does not vary and consequently $y_{it} = \sum_{s=1}^{S} y_{sit}$ and $n_{it} = \sum_{s=1}^{S} n_{sit}$. In this way the Markov model utilizes data on the infection status whilst allowing for error in reporting due to suboptimal sensitivity of each surveillance system.

Within a Bayesian framework the posterior density of the parameters are a function of the likelihood of the model and the priors of the parameters and augmented data;

$$\pi(\lambda,\gamma,\alpha,\omega) = p(\lambda)p(\gamma)p(\alpha)p(\omega)\prod_{i=1}^{M}\prod_{t=1}^{T} p(x_{it}|z_{it},\alpha)p(y_{it}|z_{it},n_{it},\omega)p(z_{it}|z_{it-1},\lambda,\gamma)$$

The model parameters were estimated using Markov chain Monte Carlo (MCMC) methods [2]. Where the surveillance sensitivity parameters (α and ω) take one value they were estimated using a Gibbs sampler where priors were assumed to be beta distributed with parameters $\alpha_1 = 2$ and $\beta_1 = 2$ in the case of α and $\alpha_2 = 2$ and $\beta_2 = 2$ in the case of ω. The augmented data $\mathbf{z}$ were updated using a Gibbs sampler based on the current values of the transition intensities, the data and values of α and ω. The posterior estimate of $\mathbf{z_i}$ was used in estimation of the false omission rate. The parameters λ and γ were estimated using the Metropolis-Hastings step and the priors for λ and γ were log-normally distributed with mean ($\lambda_m = -2$ and $\gamma_m = -2$) and variance ($\lambda_v = 0.1$ and $\gamma_v = 0.1$).

The model described above was extended the test hypotheses of variation in the sensitivity of each surveillance system. District variation in sensitivity of AFP surveillance was included by allowing α to vary by district, first assuming independence of each district and sampling estimates via a Gibbs sampler. A linear relationship between the sensitivity of α_i and the incidence of poliomyelitis k_i (on a log_{10} scale) was modelled assuming $\log\left(\frac{\alpha_i}{1-\alpha_i}\right) = g + hk_i$ where g and h were estimated using the Metropolis-Hastings step. For environmental surveillance a similar approach was taken, first assuming independence and then exploring additional functional forms. A mixed effects model was assumed where the sensitivity at each site took the form $\log\left(\frac{\omega_{si}}{1-\omega_{si}}\right) = \beta + b_i + \epsilon_{si}$ where β was the average sensitivity and was modelled assuming $\beta \sim \text{Normal}(\mu_\beta, \sigma_\beta^2)$, b_i was the district random effects which were modelled assuming $b_i \sim \text{Normal}(0, \sigma_b^2)$, and ϵ_{si} were the site random effects which were modelled assuming $\epsilon_{si} \sim \text{Normal}(0, \sigma_\epsilon^2)$. Each parameter ($\beta$, σ_b^2 and σ_ϵ^2) was estimated using a Gibbs sampler where the priors on the variances were inverse gamma distributed. In another model sensitivity was assumed to be a function of catchment size m_{si} (on a $log_{10}scale$). A cubic relationship was assumed between catchment size and sensitivity to allow for a non-linear relationship (ie. $\log\left(\frac{\omega_{si}}{1-\omega_{si}}\right) = dm_{si}^3 + cm_{si}^2 + bm_{si} + a$), where a, b, c and d were estimated, and simpler models were tested by assuming each parameter was equal to zero (ie. for a quadratic model $d = 0$). The fit of these alternative models to the data were compared using Bayes factors.

S2. Estimation of catchment area size from digital elevation modelling

During wastewater sample collection, GPS coordinates are taken of the sampling location, where in subsequent visits the same location is sampled. The catchment area of sampling locations has been estimated using a digital elevation modelling approach, where the resolution of the topography is 30x30m [3]. The topography of the landscape and river flows are used to identify land areas where wastewater is likely to flow towards the environmental sampling site. The estimated polygon is overlaid onto resolute population size estimates of Pakistan, available at a 100x100m resolution [4]. The estimates of catchment areas do not include any information regarding sewer networks in Pakistan, largely because the information is unavailable. A summary of the estimated catchment sizes (as of March 2016) are shown in S1 Table.

S3. Estimation of Bayes factors using thermodynamic integration

Bayes factors were estimated using thermodynamic integration (TI) methods, which enabled comparison of the different multistate models [5]. These methods lend themselves well to estimation of model fit in a framework with augmented data such as multistate models, because they are not reliant upon knowing the number of parameters within a model (unlike the better-known deviance information criteria). Given a model M, with parameter vector θ and applied on dataset D, the posterior probability distribution is given by Bayes Theorem;

$$p(\theta|D, M) = \frac{p(D|\theta, M)p(\theta|M)}{p(D|M)}$$

where $p(\theta|M)$ is the prior distribution, $p(D|\theta, M)$ is the likelihood function, and

$$p(D|M) = \int_{\theta} p(D|\theta, M)p(\theta|M)d\theta$$

is the normalization constant, sometimes referred to as the model evidence, that may be interpreted as the marginal likelihood of model M given the data D only. There are many different methods available to numerically sample $p(D|M)$, and owing to its general applicability and more robust statistical framework TI is used. The TI methods exploit the *power posterior* defined as follows:

$$p_{\beta}(\theta|D, M) = \frac{p(D|\theta, M)^{\beta}p(\theta|M)}{p(D|\beta, M)}$$

where $p(D|\beta, M)$ is a normalizing constant;

$$p(D|\beta, M) = \int_{\theta} p(D|\theta, M)^{\beta}p(\theta|M)d\theta$$

the gradient of the log of the power-posterior, $log(p(D|M))$ is equivalent to the expected log-likelihood of θ, where the expectation is taken over the power posterior. This can be approximated by selecting r values of β ranging from 0 to 1, estimating the power posterior and using Simpson's Rule to estimate the model evidence (equivalent to the area under the curve). Note that this estimate can become computationally intensive as each value of β requires an MCMC output of sufficient length to limit estimation error, and r needs to be sufficiently large to reduce discretization error from the values of β. In this application $r = 50$ and 100,000 iterations of the MCMC chain were used. Methods to improve the efficiency of TI approximations are an important area of research.

When two models M_0 and M_1 are being compared, the Bayes Factor refers to

$$B_{01} = \frac{p(D|M_1)}{p(D|M_0)}$$

which on a log-scale is $log(p(D|M_1)) - log(p(D|M_0))$. Values greater than 1 favor M_1, while values less than 1 favor M_2, although the cut-off chosen may vary between applications. Here simulations (described below) indicated that a cut-off of 1.00 may limit type II errors.

S4. Simulations to test the model framework

Simulated data were used to test whether the model framework was able to accurately estimate parameter values and identify the correct model. Values of ω and α were varied and 100 simulations were generated for each parameter set. The model parameters were re-estimated and the median and 95% CI were compared to the simulated values; simulations correctly re-estimating parameters included the values within the 95% CI. Variation in ω was tested using Bayes Factors between the simple model and a model that included variation in ω, if the Bayes Factors were >1.00 then there was evidence for the more complex model being a better fit to the data. These simulations were repeated 10 times for each parameter set.

Figure S1 illustrates that estimates of surveillance sensitivity were both accurate and precise for outputs from the model without covariates. A difference in environmental sensitivity (ω) between districts could be detected if the difference was >10% and if AFP sensitivity was near 50% (Table S2). For example when AFP sensitivity (α) was 50% and the difference in environmental sensitivity was greater than 20% all simulations selected the model with variation in sensitivity of environmental sampling, when the simulated difference in environmental sensitivity was less pronounced model selection was less accurate. When $\alpha = 0.1$ model selection was still able to detect a difference in sensitivity when the true difference in ES sensitivity was more than 20% but at values less than 20% there was insufficient discriminatory power to correctly identify variation in ω.

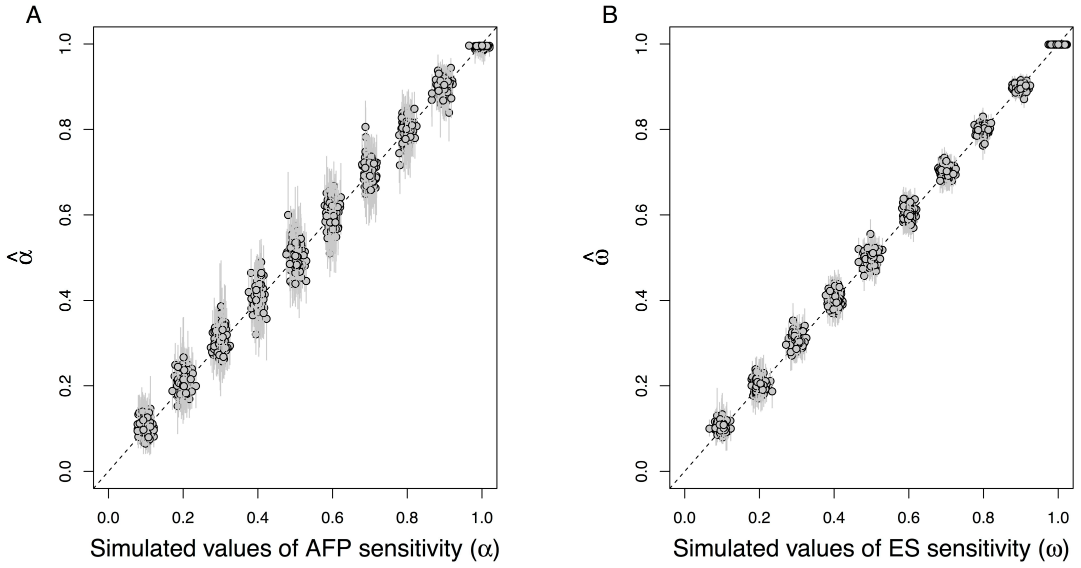

S1 Figure. Comparison of simulated (x-axis) and estimated (y-axis) values to compare the accuracy and precision of (A) AFP and (B) environmental surveillance sensitivity. For clarity the simulated values are jittered across the x-axis. The grey lines indicate the 95% credible intervals of the posterior distribution for the sensitivity parameters.

1 Tables

S1 Table. Environmental sampling sites in Pakistan. Catchment size estimates were obtained from the Novel-t website. Estimates of sensitivity are from the model assuming a mixed effects structure.

District	Site	Ref	Site Ref	Numbers of people within catchment area (March 2016)	Sensitivity estimate (95 % CrI)
DIKhan	MODC	1	PAK/KP/DIK/MD-1	2,750	33.6 (14.3 ,56)
	Sher Pao Basti Drain	2	PAK/KP/DIK/SP-1	14	35.2 (16.2 ,57.9)
	Zafar Abad Drain	3	PAK/KP/DIK/ZA-1	6,660	33.3 (14.5 ,57.2)
Peshawar	LRM, Lara Ma	1	PAK/KP/PWR/LM-1	29,159	63.3 (51.3 ,74.1)
	Shaheen town	2	PAK/KP/PWR/ST-1	39,105	79.2 (69 ,87.6)
Islamabad	Sabzi Mandi	1	PAK/IB/CDA/SM-1	457,953	58.7 (29.5 ,81.5)
Rawalpindi	Dhok Dalal	1	PAK/PB/RWP/DD-1	60,339	64.6 (43.3 ,82.7)
	Safdar abad (Hazara Colony)	2	PAK/PB/RWP/SA-1	85,757	72.3 (58 ,83.8)
Lahore	Gulshan-e-Ravi PS	1	PAK/PB/LHR/GR-1	458	47.7 (33.4 ,62)
	Multan road Disposal Station	2	PAK/PB/LHR/MR-1	14,470	54.2 (39.4 ,67.5)
	Main Outfall PS	3	PAK/PB/LHR/OF-1	117,615	57 (45.3 ,68.1)
Faisalabad	PS-3	1	PAK/PB/FSD/GM-1	241,924	43.2 (21.8 ,68.6)
	PS-27	2	PAK/PB/FSD/GM-2	204,014	36.9 (15.8 ,61.3)
	Ismail City Road	3	PAK/PB/FSD/IR-1	4,638	35.2 (14.7 ,60.2)
Multan	Ali Town	1	PAK/PB/MUL/AT-1	47,379	52.4 (35.1 ,68.5)
	Kotla Abdul Fatah	2	PAK/PB/MUL/KF-1	703,134	49.8 (31.8 ,66.2)
	Suraj Miani	3	PAK/PB/MUL/SM-1	564,875	54.6 (37.8 ,71.1)
Sukkur	Makka PS	1	PAK/SD/SUK/NS-1	300,865	52.3 (34.4 ,69.4)
	Miani PS Taluka	2	PAK/SD/SUK/SC-1	165,083	52.9 (35.1 ,71.9)
Hyderabad	Tulsidas PS	1	PAK/SD/HYD/HC-1	96	76.1 (61.3 ,88.7)
Karachi: Baldia	Sajjan Goth	1	PAK/SD/KHI/BD-1	316,094	50.2 (35.6 ,66.4)
	Composite Site	2	PAK/SD/KHI/BD-3	47,537	57.5 (41.9 ,71.5)
Karachi: Gadap	Machhar Colony	1	PAK/SD/KHI/GP-1	236,028	63.3 (50.8 ,75.5)
	Sohrab Goth	2	PAK/SD/KHI/GP-2	58,922	71.5 (58.3 ,82)
	Khamiso Goth	3	PAK/SD/KHI/GP-3	236,028	44.4 (28.1 ,60.7)
Karachi: Gulshan-e-Iqbal	Chakora Nulla	1	PAK/SD/KHI/GI-1	119,878	46.3 (32.6 ,60.2)
	Rashid Minhas road	2	PAK/SD/KHI/GI-2	467,832	63.2 (48.3 ,75.7)
Quetta	Jam-e-Salfia	1	PAK/BN/QTA/JS/1	51,080	44.9 (30.6 ,58.5)
	Killi Jatak and Takhthani By-Pass	2	PAK/BN/QTA/JT/1	17,019	64.2 (50.7 ,77)
	Surpul	3	PAK/BN/QTA/SP-1	744,173	50.8 (34.2 ,67.2)
Killa Abdullah	Army Kaziba	1	PAK/BN/KAB/AK-1	6,169	54.7 (31.4 ,74.1)
	Hadi Packet	2	PAK/BN/KAB/HP-1	241	45.6 (25 ,67.6)

S2 Table. Simulations to test detection of a difference in environmental sampling sensitivity between districts. Different values of AFP and ES sensitivity were assumed and used to simulate data (10 datasets per scenario), parameters were estimated using a model with one ES parameter and a model where ES sensitivity varied between districts, and Bayes Factors were used to compare model fits. In all simulations the rate of infection (λ) was 0.1 and the rate of recovery (γ) was 1/6 months. Correct identification of the extended model was when the Bayes factor was $\geq$1.00.

Sensitivity of AFP (α)	Sensitivty ES1 (ω_1)	Sensitivty ES2 (ω_2)	% simulations correctly identifying ES variation	Average Bayes factor
0.1	0.5	0.1	70	7.03
0.1	0.5	0.2	100	7.56
0.1	0.5	0.3	20	-2.42
0.1	0.5	0.4	0	-12.79
0.5	0.5	0.1	100	57.65
0.5	0.5	0.2	100	30.04
0.5	0.5	0.3	50	0.12
0.5	0.5	0.4	0	-12.43

S3 Table. Bayes factors for each model applied to AFP and environmental surveillance data of poliovirus in Pakistan, January 2011 to August 2015. A Bayes factor greater than 1.00 indicates an improved model fit when compared to the baseline model. The starred models have an improved fit to the data in comparison to the simplest model and the overall best-fitting model is highlighted in bold.

**Model Assumption for AFP surveillance**	**Assumption for environmental surveillance**	**Number of parameters**	**Model evidence**	**Bayes Factor**
One value	One value	4	-481.5	NA
Independent values per district	One value	17	-508.2	-26.7
Linear increase with log10(incidence)	One value	5	-426.8	54.6*
One value	Independent values per district	17	-575.0	-93.5
Independent values per district	Independent values per district	30	-591.9	-110.4
Linear increase with log10(incidence)	Independent values per district	18	-514.7	-33.2
One value	Linear increase with catchment size	5	-485.2	-3.7
Independent values per district	Linear increase with catchment size	18	-508.8	-27.3
Linear increase with log10(incidence)	Linear increase with catchment size	6	-432.0	49.5*
One value	Mixed effects structure	5	-575.9	-94.5
Independent values per district	Mixed effects structure	18	-592.6	-111.2
Linear increase with log10(incidence)	Mixed effects structure	6	-516.7	-35.3

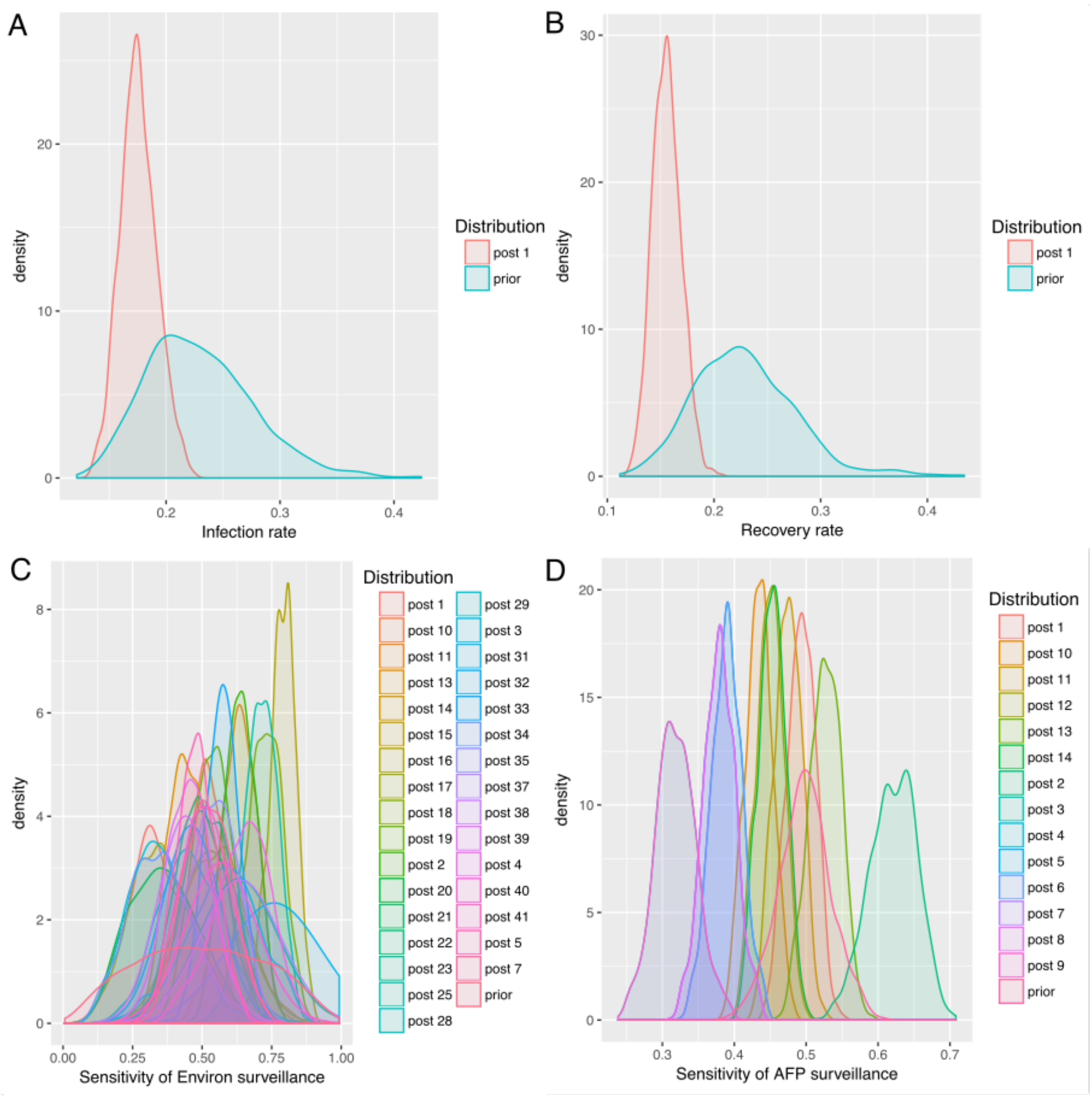

S2 Figure. Prior and posterior distributions for each of the parameters in the best-fitting model A) The infection rate (λ) B) the recovery rate (γ) C) Sensitivity of environmental surveillance (ω) for each sampled site D) Sensitivity of AFP surveillance (α) for each district.

References

[1] Gentleman, R. C., Lawless, J. F., Lindsey, J. C. & Yan, P. Multi-state Markov models for analysing incomplete disease history data with illustrations for HIV disease. *Statistics in medicine* **13**, 805–21 (1994). URL http://www.ncbi.nlm.nih.gov/pubmed/7914028.

[2] Robert, C. & Casella, G. *Introducing Monte Carlo Methods with R*, vol. 83 (Springer New York, New York, NY, 2010), 1st editio edn. URL http://link.springer.com/10.1007/978-1-4419-1576-4.

[3] Novel-t. Catalogue of Environmental Sites Supporting Polio Eradication (2016). URL http://maps.novel-t.ch/#/catalog/all.

[4] Gaughan, A. E., Stevens, F. R., Linard, C., Jia, P. & Tatem, A. J. High resolution population distribution maps for Southeast Asia in 2010 and 2015. *PloS one* **8**, e55882 (2013). URL http://www.pubmedcentral.nih.gov/articlerender.fcgi?artid=3572178{&}tool=pmcentrez{&}rendertype=abstract.

[5] Lartillot, N. & Philippe, H. Computing Bayes factors using thermodynamic integration. *Systematic biology* **55**, 195–207 (2006). URL http://www.ncbi.nlm.nih.gov/pubmed/16522570.
